# PDGFA/PDGFRα-regulated GOLM1 promotes human glioma progression through activation of AKT

**DOI:** 10.1186/s13046-017-0665-3

**Published:** 2017-12-28

**Authors:** Ran Xu, Jianxiong Ji, Xin Zhang, Mingzhi Han, Chao Zhang, Yangyang Xu, Yuzhen Wei, Shuai Wang, Bin Huang, Anjing Chen, Di Zhang, Qing Zhang, Wenjie Li, Zheng Jiang, Jian Wang, Xingang Li

**Affiliations:** 1 Department of Neurosurgery, Qilu Hospital of Shandong University and Brain Science Research Institute, Shandong University, #107 Wenhua Xi Road, Jinan, 250012 China; 2 Department of Biomedicine, University of Bergen, Jonas Lies vei 91, 5009 Bergen, Norway; 3 Department of Neurosurgery, Jining No.1 People’s Hospital, Jiankang Road, Jining, 272011 China

**Keywords:** AKT, GOLM1, Glioma, PDGFA, Progression

## Abstract

**Background:**

Golgi Membrane Protein 1 (GOLM1), a protein involved in the trafficking of proteins through the Golgi apparatus, has been shown to be oncogenic in a variety of human cancers. Here, we examined the role of GOLM1 in the development of human glioma.

**Methods:**

qRT-PCR, immunohistochemistry, and western blot analysis were performed to evaluate GOLM1 levels in cell lines and a cohort of primary human glioma and non-neoplastic brain tissue samples. Glioma cell lines were modified with lentiviral constructs expressing short hairpin RNAs targeting GOLM1 or overexpressing the protein to assess function in proliferation, viability, and migration and invasion in vitro using EdU, CCK8, clone-forming, Transwell assays, 3D tumor spheroid invasion assay and in vivo in orthotopic implantations. Protein lysates were used to screen a membrane-based antibody array to identify kinases mediated by GOLM1. Specific inhibitors of PDGFRα (AG1296) and AKT (MK-2206) were used to examine the regulation of PDGFA/PDGFRα on GOLM1 and the underlying pathway respectively.

**Results:**

qRT-PCR, immunohistochemistry and western blot analysis revealed GOLM1 expression to be elevated in glioma tissues and cell lines. *S*ilencing of GOLM1 attenuated proliferation, migration, and invasion of U251, A172 and P3#GBM (primary glioma) cells, while overexpression of GOLM1 enhanced malignant behavior of U87MG cells. We further demonstrated that activation of AKT is the driving force of GOLM1-promoted glioma progression. The last finding of this research belongs to the regulation of PDGFA/PDGFRα on GOLM1, while GOLM1 was also a key element of PDGFA/PDGFRα-mediated activation of AKT, as well as the progression of glioma cells.

**Conclusions:**

PDGFA/PDGFRα-regulated GOLM1 promotes glioma progression possibly through activation of a key signaling kinase, AKT. GOLM1 interference may therefore provide a novel therapeutic target and improve the efficacy of glioma treatment, particularly in the case of the proneural molecular subtype of human glioma.

**Electronic supplementary material:**

The online version of this article (10.1186/s13046-017-0665-3) contains supplementary material, which is available to authorized users.

## Background

Glioblastoma multiforme (GBM) is the most aggressive form of human glioma and is highly resistant to therapy in part due to its highly infiltrative growth [1]. The median survival time of GBM patients remains at a mere 9–12 months despite multimodal treatment, which includes surgery, radio- and chemotherapy [2, 3]. Therefore, novel strategies which can attenuate the malignant proliferation and infiltration of GBM are desperately needed.

Molecular profiling of primary tumors has led to the identification of critical pathways involved in the development of human glioma. The hope is that this approach will lead to more effective targeted molecular therapies. Studies have recently implicated Golgi proteins in the development of human gliomas. Expression of Golgi phosphoprotein 3 (GOLPH3), for example, has been associated with worse prognosis in human glioma patients and furthermore was shown to promote glioma progression [4]. GOLM1, also known as GP73 and GOLPH2, is a highly-phosphorylated protein located in the cis and medial-Golgi apparatus [5]. GOLM1 processes proteins synthesized in the rough endoplasmic reticulum and assists in the transport of protein cargo through the Golgi apparatus [6]. Furthermore, GOLM1 has been identified as a serum marker for hepatocellular carcinoma [7]. An increasing number of studies have also revealed GOLM1 as a promoter of proliferation, invasion, and migration in diverse human cancers, including hepatocellular carcinoma, prostate cancer [8], oesophageal cancer [9], gastric cancer [10], cutaneous melanoma [11].

Studies have also demonstrated that GOLM1 can be upregulated by platelet derived growth factor (PDGF) and activate critical downstream signaling kinases such as AKT, ERK, and S6 K in the hepatocellular cell line Huh7 [12]. These results implicated a role for GOLM1 in the development of PDGF-mediated hepatocellular carcinoma. PDGF signaling also has critical roles in the process of normal brain development [13]. Furthermore, the gene for platelet derived growth factor receptor alpha (PDGFRα) is one of the most frequently amplified genes in clinical GBM samples [13].

Here, we examined the role of GOLM1 in the development of human glioma and its functional relationship with PDGFRα. TCGA and Rembrandt databases and an independent cohort of primary glioma samples were analyzed for expression levels of *GOLM1* based on molecular subtype (classical, mesenchymal, proneural, and neural) to reveal an association with PDGFRα. Functional assays in glioma cell lines confirmed this association and illuminated a potential role for GOLM1 in PDGFRα signaling. Therefore, we identified GOLM1 as a potential oncogene in the development of human glioma and target in the treatment of the disease.

## Methods

### Ethics statement

Experiments were approved by the Research Ethics Committee of Qilu Hospital of Shandong University (Jinan, China), and written informed consent was obtained from all participating individuals. All surgical interventions and post-operative animal care were approved by Institutional Animal Care and Use Committee (IACUC) of Shandong University.

### Cell culture and chemicals

The cell population derived from normal human astrocytes (NHA) and P3#GBM cells were kind gift from the Department of Biomedicine at the University of Bergen (Bergen, Norway). U87MG, U251 and A172 were obtained from the Culture Collection of the Chinese Academy of Sciences (Shanghai, China). P3#GBM cells were cultured in serum-free Neurobasal medium (Gibco, USA) supplemented with 2% B27 Neuro Mix (Thermo Fisher Scientific, USA), 20 ng/mL epidermal growth factor (EGF; Thermo Fisher Scientific, USA), and 10 ng/mL basic fibroblast growth factor (bFGF; PeproTech, USA). GBM and NHA cells were cultured in Dulbecco’s modified Eagle’s medium (DMEM; Life Technologies-Thermo Fisher Scientific, USA) supplemented with 10% fetal bovine serum (FBS; Life Technologies-Thermo Fisher Scientific, USA) and maintained at 37 °C in a humidified chamber containing 5% CO_2_. Recombinant human PDGF-AA (Peprotech, USA) was dissolved in phosphate buffered saline (PBS), and AG-1296, an inhibitor of PDGFRα (Selleck, China), was dissolved in DMSO before addition to media. The small molecule MK-2206 (Apexbio, USA) was dissolved in DMSO and used as an inhibitor of AKT phosphorylation.

### Quantitative real-time PCR (qRT-PCR)

Total RNA was isolated from the cells using Trizol reagent (Takara, Japan) according to the manufacturer’s protocol. Total RNA (1 μg) was reverse-transcribed, and the resulting cDNA was used as a template in qRT-PCR using a standard SYBR premix Ex Taq (Takara, Japan) on the Real-Time PCR Detection System (Roche, 480II, USA). *GAPDH* served as the internal control, and experiments were conducted in triplicate. The following primers were used: *GAPDH* forward, 5’-AATGAAGGGGTCATTGATGG-3′, reverse, 5’-AAGGTGAAGGTCGGAGTCAA-3′; *GOLM1* forward 5′-CCGGAGCCTCGAAAAGAGATT-3′, reverse 5′-ATGATCCGTGTCTGGAGGTC-3′.

### Western blotting analysis

Cells and tissues were lysed in RIPA buffer (Pierce-Thermo Fisher Scientific, USA) containing a protein inhibitor cocktail. Protein concentrations were quantified using Pierce Protein Assay Kit (Pierce, USA). Proteins (20 μg) were separated by SDS-PAGE, and detected by primary antibodies for GOLM1(1:500; Abcam), GSK3β (phospho S9) (1:5000; Abcam), GSK3β (1:2000; Abcam), phospho-p44/42 MAPK (ERK1/2) (Thr202/Tyr204) (1:1000; Cell Signaling Technology; Danvers, MA, USA), p44/42 MAPK (ERK1/2) (1:1000; Cell Signaling Technology), phospho-AKT (Ser473) (1:1000; Cell Signaling Technology), Snail (1:1000; Abcam), ZEB1(1:1000; Abcam), AKT (pan) (1:1000; Cell Signaling Technology), phospho-PDGF receptor α (Tyr754) (1:1000; Cell Signaling Technology) and GAPDH (1:2000; Cell Signaling Technology). Proteins were quantified using chemiluminescence (Bio-Rad, USA) according to the manufacturer’s protocol.

### Construction of stably transfected cells

Lentiviral constructs containing full length GOLM1 (Lenti-GOLM1; GeneChem Technologies; Shanghai, China) or short hairpin RNAs (sh-GOLM1–1, sh-GOLM1–2; GeneChem Technologies) were used to generate stable GOLM1 overexpressing or knockdown cell lines. U251, A172 and P3#GBM cell lines were infected with sh-GOLM1–2, while U87MG cells were infected with Lenti-GOLM1. After 48 h, U87MG, U251, A172 and P3#GBM cells were exposed to puromycin (0.5 μg/mL, 2 μg/mL, 2 μg/mL and 2 μg/mL respectively; A1113802; Gibco-Thermo Fisher Scientific) for an additional 2 weeks to enrich for cells harboring the constructs. The targeting sequences in the shRNAs were the following: sh-NC 5’-TTCTCCGAACGTGTCACGTtt-3′; sh-GOLM1–1 5′- GTGGCTTAGAATTTGAACAtt-3′; sh-GOLM1–2 5′- CAAGCTGTACCAGGACGAAtt-3′.

### Migration and invasion assays.

Invasion and migration of U87MG, U251 and A172 cells were evaluated in uncoated and matrigel-coated (BD Biosciences; Bedford, MA, USA) Transwell chambers (8 μm pores; Corning Costar; Oneonta, NY, USA). Cells (2 × 10^4^) were seeded in the top chamber in DMEM (200 μL) with 1% FBS and the lower chamber was filled with DMEM (600 μL) containing 30% FBS. Transwell chambers were incubated for 24 h. Cells that had invaded or migrated into the lower surface were fixed with 4% paraformaldehyde (Solarbio; Beijing, China), stained with crystal violet (Solarbio) for 20 min, and counted under bright field microscopy. Images were acquired from 5 random fields in each well, and cell numbers were determined using Kodak MI software. Each experiment was performed in triplicate.

For 3D spheroid invasion assay, spheroids were generated through incubating P3#GBM cells in the spheroid formation matrix for 96 h in a 3D culture qualified 96-well spheroid formation plate. Spheroids were embedded into the invasion matrix (Trevigen, USA) composed of basement membrane proteins in the 96-well plate. Glioma spheroids were photographed every 48 h under Leica microscope. The spheroid at 0 h was used as a reference point for measurement of the invaded area.

### Immunofluorescence staining (IF)

Cells were cultured on coverslips, fixed with 4% paraformaldehyde, permeabilized with 0.4%Triton X-100, blocked with 5% bovine serum albumin, and incubated with primary antibody against GOLM1 (1:200; Abcam) or TRA1–85 (1:200; R&D Systems) at 4 °C overnight. Primary antibody was subsequently detected with an Alexa Fluor 594 conjugated goat anti-rabbit IgG antibody (1:800; Abcam) or Alexa Fluor 488 conjugated goat anti-mouse IgG antibody (1:800; Abcam) respectively. The cytoskeleton was visualized through staining with anti-stain 488 phalloidin (Cytoskeleton; Denver, CO, USA) according to manufacturer’s instructions. Cell nuclei were stained with DAPI (Sigma-Aldrich; Hamburg, Germany). Images for analysis were obtained under fluorescence microscopy (Leica; Wetzlar, Germany).

### Immunohistochemistry (IHC)

Tumor specimens were obtained from glioma patients (*n* = 69; WHO grade II-IV) who had undergone surgery at the Department of Neurosurgery in Qilu Hospital of Shandong University. Non-neoplastic brain tissue samples (*n* = 6) were collected from partial resections of normal brain as decompression treatment for severe head injuries.

Paraffin blocks were sectioned and citrate/heat antigen retrieval was performed. IHC staining was performed using the ABC kit (Origene; Rockville, MD) and visualized using the DAB kit (ZSGB-Bio; Beijing, China) according to the manufacturer’s instructions. The following primary antibodies were used: GOLM1 (1:500; Abcam), Ki-67 (1:500; Abcam) and phospho-PDGF receptor α (Tyr754) (1:500; Cell Signaling Technology). IHC staining was scored as follows: 0, no staining; 1, weak staining in <50% cells; 2, weak staining in ≥50% cells; 3, strong staining in <50% cells; and 4, strong staining in ≥50% cells.

### EdU assay

EdU assay was performed using the EdU Apollo 567 Cell Tracking Kit (Rib-bio; Guangzhou, China). Treated and control cells (2 × 10^4^/well) were seeded onto 24-well plates and incubated with 5-ethynyl-20-deoxyuridine (EdU; 200 μM) for 2 h at 37 °C. Cells were fixed with 4% paraformaldehyde for 20 min, treated with 0.5% Triton X-100 for 10 min, rinsed with PBS three times, and incubated with 100 μL of Apollo reagent for 30 min. Nuclei were labeled with Hochest 33342. The percentage of EdU-positive cells was calculated based on counts from 500 cells in three independent experiments.

### Cell viability assay

Cell viability was assessed using the Cell Counting Kit-8 assay (CCK-8) according to the manufacturer’s protocol (Dojindo; Tokyo, Japan). Cells were seeded at 2 × 10^3^ cells /well in 96-well plates and incubated at 37 °C for 24, 48, and 72 h in a humidified chamber containing 5% CO_2_. CCK-8 solution (10 μL) was added to each well, and the plates were incubated for 1 h at 37 °C. The absorbance of cells at 450 nm (OD450) was measured in a microplate reader (Bio-Rad, USA).

### Colony forming assay

Cells (120 cells/well) were seeded onto six-well plates, and the medium was changed thereafter twice each week. After 2 weeks, cells were fixed in 100% methanol and stained with 5% crystal violet. Colonies of more than 50 cells were counted. Data reported represent the average of three independent experiments.

### Phospho-kinase array

Proteins lysates were incubated with a human phospho-kinase array (membrane-based antibody array; R&D Systems, USA) for the parallel determination of the relative levels of human protein kinase phosphorylation (*n* = 43 kinase phosphorylation sites) and related total proteins (*n* = 2). Analysis of the phospho-kinase array was performed using chemiluminescence according to the manufacturer’s instructions, and pixel densities of the spots were analyzed using Image J software.

### Animal studies

For generation of orthotopic xenografts, 4-week-old nude male nude mice (SLAC laboratory animal Center, Shanghai, China) were maintained in a barrier facility on high-efficiency particulate air (HEPA)-filtered racks. Animals (*n* = 48) were divided equally among 6 groups (*n* = 8 to each group): U251-NC, U251-sh-GOLM1, P3#GBM-NC, P3#GBM-sh-GOLM1, U87MG-Lenti-NC and U87MG-Lenti-GOLM1. Cells (1 × 10^6^) were implanted into mice brains using a stereotactic apparatus (KDS310, KD Scientific; Holliston, MA, USA). Animals were closely followed and euthanized by cervical dislocation when they exhibited symptoms, such as severe hunchback posture, apathy, decreased motion or activity, dragging legs, or drastic loss of body weight. Tumors were excised, formalin-fixed, paraffin-embedded, and sectioned for hematoxylin and eosin (HE) staining and IHC.

For subcutaneous GBM model, nude mice (*n* = 30) were divided into six groups (U87MG-Lenti-NC, U87MG-Lenti-GOLM1, U251-NC, U251-sh-GOLM1, P3#GBM-NC, P3#GBM-sh-GOLM1, 5 mice per group). Cells were harvested by trypsinization, resuspended at 10^7^ cells/mL in a 1:1 solution of PBS/Matrigel (BD Biosciences, USA), and injected subcutaneously into the right shoulder of the mouse. The tumor tissues were isolated 30 days after injection. Primary tumors were measured in 3 dimensions (a, b, c), and volume was calculated as abc × 0.52 [14].

### Bioinformatic analysis

Molecular array data used for analysis was obtained from publicly available datasets, the TCGA (http://cancergenome.nih.gov/) and Rembrandt (http://www.betastasis.com/glioma/rembrandt/).

### Statistical analysis

All data are presented as the mean ± the standard error of the mean (S.E.M). The Student’s t-test was used only when two groups were being compared. Analysis of variance (ANOVA) was used in cases where there were more than two groups being compared. Survival curves were estimated by the Kaplan-Meier method and compared using the log-rank test. A two-tailed χ2 test was used to determine the association between GOLM1 and phospo-PDGFRα (Tyr 754). GraphPad Prism version 7.00 software program (GraphPad; La Jolla, CA, USA) was used to analyze in vitro and in vivo experiments. Differences with *P*-values <0.05 were considered statistically significant.

## Results

### *GOLM1* expression is elevated in human gliomas.

**Fig. 1:**
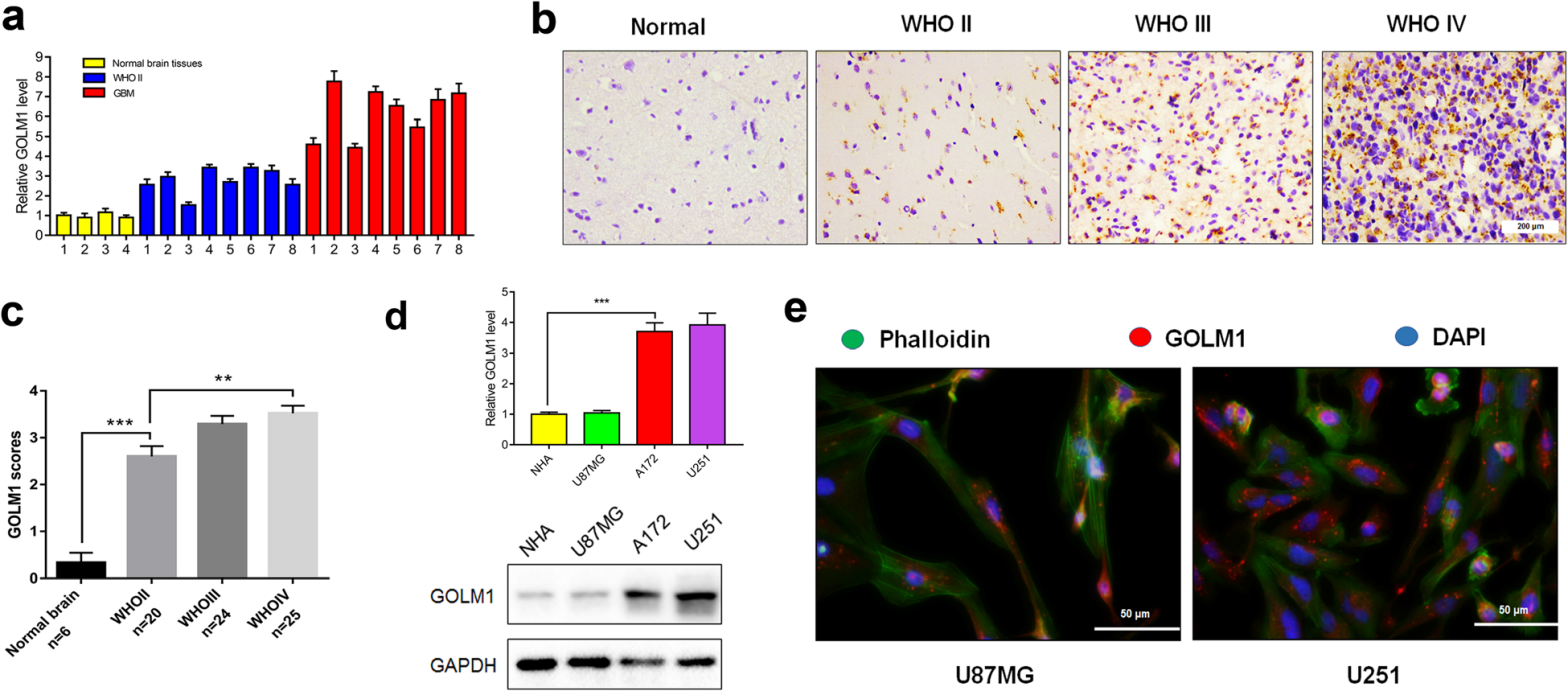
*GOLM1* expression is elevated in human gliomas. **a** qRT-PCR to measure relative expression levels of *GOLM1* in different grade glioma and non-neoplastic tissue samples. **b** Representative images of IHC staining for GOLM1 in human glioma and non-neoplastic brain tissue samples. Scale bar = 200 μm; **c** Graphic representation of scoring performed on IHC staining for GOLM1 in glioma and non-neoplastic tissue samples. Bar graphs show the mean ± the standard error of the mean (SEM) for each group. **d** qRT-PCR (upper) and Western blot analysis (lower) of GOLM1 levels in NHA, U87MG, A172 and U251 cells. **e** Representative images of immunofluorescence staining performed for GOLM1 (red) in U87MG and U251 cells and visualized under fluorescence microscopy. F-actin was stained with phalloidin (green). Nuclei were stained with DAPI (blue). Scale bar = 50 μm. (**P < 0.01, ***P < 0.001)

To investigate the function of GOLM1 in glioma, we first examined levels of *GOLM1* mRNA in primary human gliomas (WHO grade II, *n =* 8; WHO grade IV, *n =* 8). Levels of *GOLM1* were elevated in human gliomas, especially in WHO grade IV gliomas (~ 5–7×), when compared to non-neoplastic brain tissue samples (*n =* 4; Fig. 1a). IHC was performed on primary tumor sections to determine whether protein levels were correspondingly increased in tumor tissues. GOLM1 was highly expressed (scores ≥3) in 9/20 low grade gliomas (LGG; 45.0%) and 40/49 high grade gliomas (HGG; 81.6%) whereas GOLM1 was nearly undetectable in non-neoplastic brain tissue samples (*n* = 6; Fig. 1b and c; Table 1). Western blot analysis performed in normal brain tissues and different grade of glioma tissues further confirmed this result (Additional file 1: Figure S1). Increased GOLM1 expression thus correlated with increasing tumor grade both at the mRNA and protein levels (*P* < 0.05, Fig. 1a and c).

**Table 1:** Correlations of GOLM1 expression with preoperative clinicopathological features in glioma patients

Variables	*n*	GOLM1 in IHC		*P* Value
		Low	High	
Age (year)				
< 55 ≥ 55	33 36	15 19	18 17	0.543
Sex				
Male Female	38 31	21 19	17 12	0.614
Tumor size				
< 4 cm ≥ 4 cm	29 40	13 23	16 17	0.298
Cystic change				
Absent Present	21 48	12 22	9 26	0.387
Edema				
None to mild Moderate to severe	31 38	14 20	17 18	0.537
WHO grade				
II III + IV	20	11	9	0.002
	49	9	40	

GOLM1 was also increased in glioma cell lines compared to NHA based on qRT-PCR and western blot analysis. GOLM1 mRNA levels were ~ 3× higher and protein levels in U251 and A172 cells were 2–3× higher compared to NHA (Fig. 1d). However, GOLM1 was not increased in U87MG compared with NHA (Fig. 1d). To confirm the results of western blot and to localize the protein, we performed IF for GOLM1 in U87MG and U251 glioma cells (Fig. 1e). GOLM1 was mainly expressed in the cytoplasm of U87MG and U251 cells, but the intensity of the GOLM1 signal was increased in U251 compared to U87MG cells (Fig. 1e).

### *GOLM1* knockdown inhibits glioma progression in U251 and A172 cells in vitro*.*

**Fig. 2:**
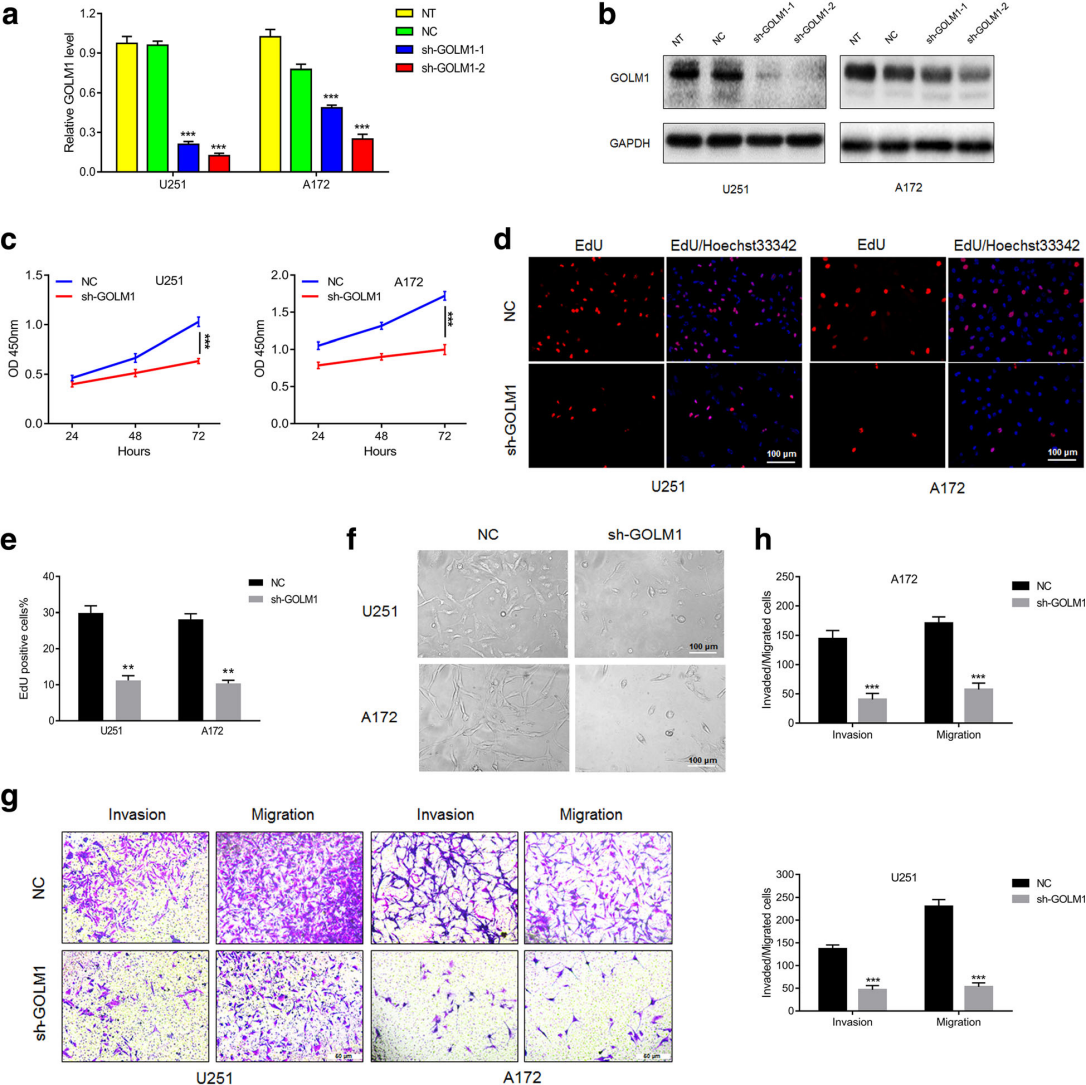
*GOLM1* knockdown inhibits glioma progression in U251 and A172 cells in vitro*.* Knockdown efficiency of lentiviral shRNA constructs, sh-GOLM1–1 and sh-GOLM1–2 in U251 and A172 cells assessed with **a** qRT-PCR and **b** western blot analysis. U251- and A172-NC and -sh-GOLM1 cell lines evaluated in **c** CCK8 assay for cell viability. **d** EdU (red) assays for proliferation rate. Nuclei are stained with DAPI (blue). Scale bar = 100 μm. **e** Graphic representation of ratios of EdU positive cells in U251- and A172-NC and sh-GOLM1 cells. Data are presented as the mean ± SEM. **f** Representative images of the morphology of U251- and A172-NC and sh-GOLM1 cells under bright field microscopy. Scale bar = 100 μm. **g** Images of Transwell assays performed with U251- and A172-NC and sh-GOLM1 expressing cells. Scale bar = 50 μm. **h** Quantification of invaded and migrated cells in Transwell assays after incubation for 24 h. Data are presented as the mean ± SEM. (**P < 0.01, ***P < 0.001)

Based on the increased expression of GOLM1 in U251 and A172 cell lines relative to U87MG cells, knockdown experiments were performed in these cells to investigate the impact of the loss of function of GOLM1 in the development of human glioma. The knockdown efficiency of two shRNA constructs was first examined in U251 and A172 cells by qRT-PCR and western blot (Fig. 2a and b). By both qRT-PCR and western blot analysis, sh-*GOLM1*–2 led to decreases in GOLM1 in both U251 and A172 cells of ~ 3×. Therefore, this lentiviral construct sh-*GOLM1*–2 (sh-GOLM1) due to increased knockdown efficiency relative to sh-*GOLM1*–1 was chosen for the development of cell populations that stably expressed the shRNA.

Growth curves for U251-sh-GOLM1 and A172-sh-GOLM1 and control cells were developed using results from the CCK8 assay. Cell growth was significantly decreased in both cell lines expressing sh-GOLM1 (Fig. 2c). Decreased proliferation upon loss of GOLM1 was further confirmed in U251 and A172 glioma cells by labeling cells with EdU (Fig. 2d and e). The number of EdU positive cells decreased from ~30% to 10% (Fig. 2e). GOLM1 knockdown thus attenuated cell viability and proliferation of U251 and A172 cells (Fig. 2c and e). The results of clone-forming assays performed in U251 and A172 -NC and -sh-GOLM1 cells further confirmed this phenomenon (Additional file 2: Figures S2a, 2b).

We also noticed that the morphology of U251 and A172 cells had changed with knockdown of *GOLM1* (Fig. 2f). Parental cells appeared more fibroblastic than modified cells which could be consistent with migration and invasion capabilities. Therefore, *GOLM1* knockdown on migration and invasion of glioma cells were investigated. In Transwell assays, migration and invasion of U251-sh-GOLM1 and A172- sh-GOLM1 cells were decreased ~ 3–5× relative to control cells (Fig. 2g and h). These results indicated that GOLM1 may act as an oncogene in glioma progression.

### Silencing of *GOLM1* inhibits invasive growth of U251 cells in vivo*.*

**Fig. 3:**
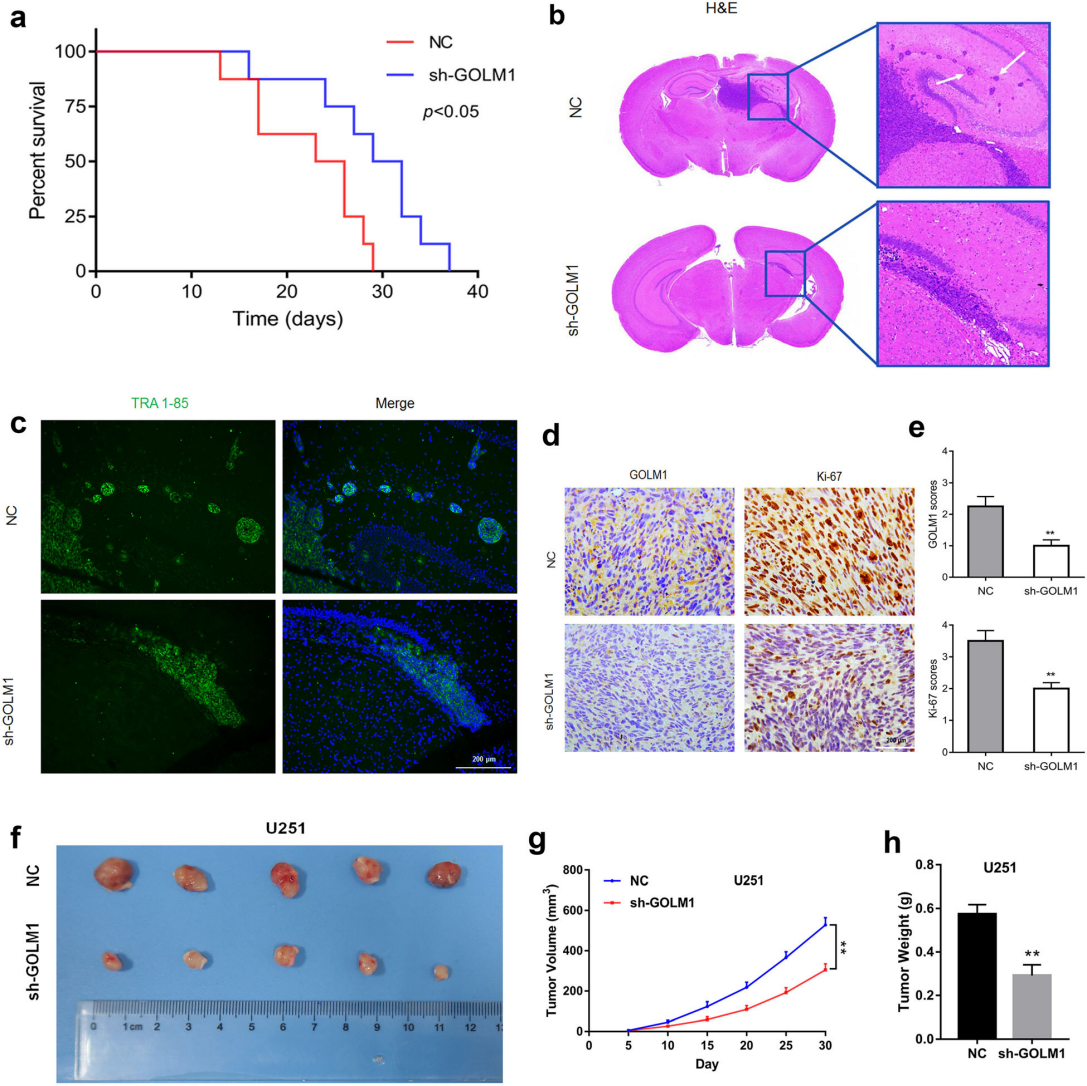
Silencing of *GOLM1* inhibits invasive growth of U251 cells in vivo*.* **a** Kaplan-Meier survival analysis of mice implanted with U251-NC (*n* = 8) and -sh-GOLM1 (*n* = 8) cells. The log-rank test was used to calculate *P*-values, which were <0.05. **b** Representative H&E images of intracranial tumors derived from U251-NC and -sh-GOLM1 cells. White arrows in zoomed image highlight tumor cells that have invaded to adjacent brain tissues. **c** Representative images of IF performed on U251-NC and -sh-GOLM1 tumors in mouse brains with TRA-1-85 antibody (green). Nuclei were labeled with DAPI (blue), and images were merged. Scale bar = 200 μm. **d** Representative IHC images of GOLM1 and Ki-67 expression in xenografts derived from the cells indicated. Scale bar = 200 μm. **e** Graphic representation of IHC scoring of GOLM1 and Ki-67 expression in brain sections derived from U251-NC and -sh-GOLM1 cells. **f** Representative images of subcutaneous U251-NC and -sh-GOLM1 xenografts after surgical removal are also shown. **g** Tumor growth curves in nude mice from the U251-NC and -sh-GOLM1 groups. **h** Tumor weight from the U251-NC and -sh-GOLM1 groups. Data are presented as the mean ± SEM. (**P < 0.01)

Modified cell lines were also orthotopically implanted in mice to investigate how *GOLM1* knockdown influenced proliferation, migration and invasion of glioma cells in vivo. Survival time of nude mice injected with U251-sh-GOLM1 cells was significantly prolonged relative to mice injected with U251-NC control cells (*P* < 0.05; Fig. 3a). On histologic examination, U251-NC tumors were larger and more invasive, and many distant satellite lesions had been generated in the peritumoral brain parenchyma (Fig. 3b). Satellite lesions were confirmed by IF staining for TRA-1-85/CD147 which is a human-specific antigen [15] (Fig. 3c).

IHC staining for the proliferation marker Ki-67 further confirmed an oncogenic role for GOLM1 in glioma [16–18]. Fewer cells were Ki-67 positive in U251-sh-GOLM1 relative to control tumors (Fig. 3d and e), indicating that loss of GOLM1 inhibited glioma proliferation in vivo. Silencing of GOLM1 also decreased volume and weight of tumor mass implanted subcutaneously, which further confirmed the inhibition of GOLM1 on glioma growth (Fig. 3f-h).

### ***GOLM1*** knockdown inhibits glioma progression in P3#GBM cells in vitro and in vivo

**Fig. 4:**
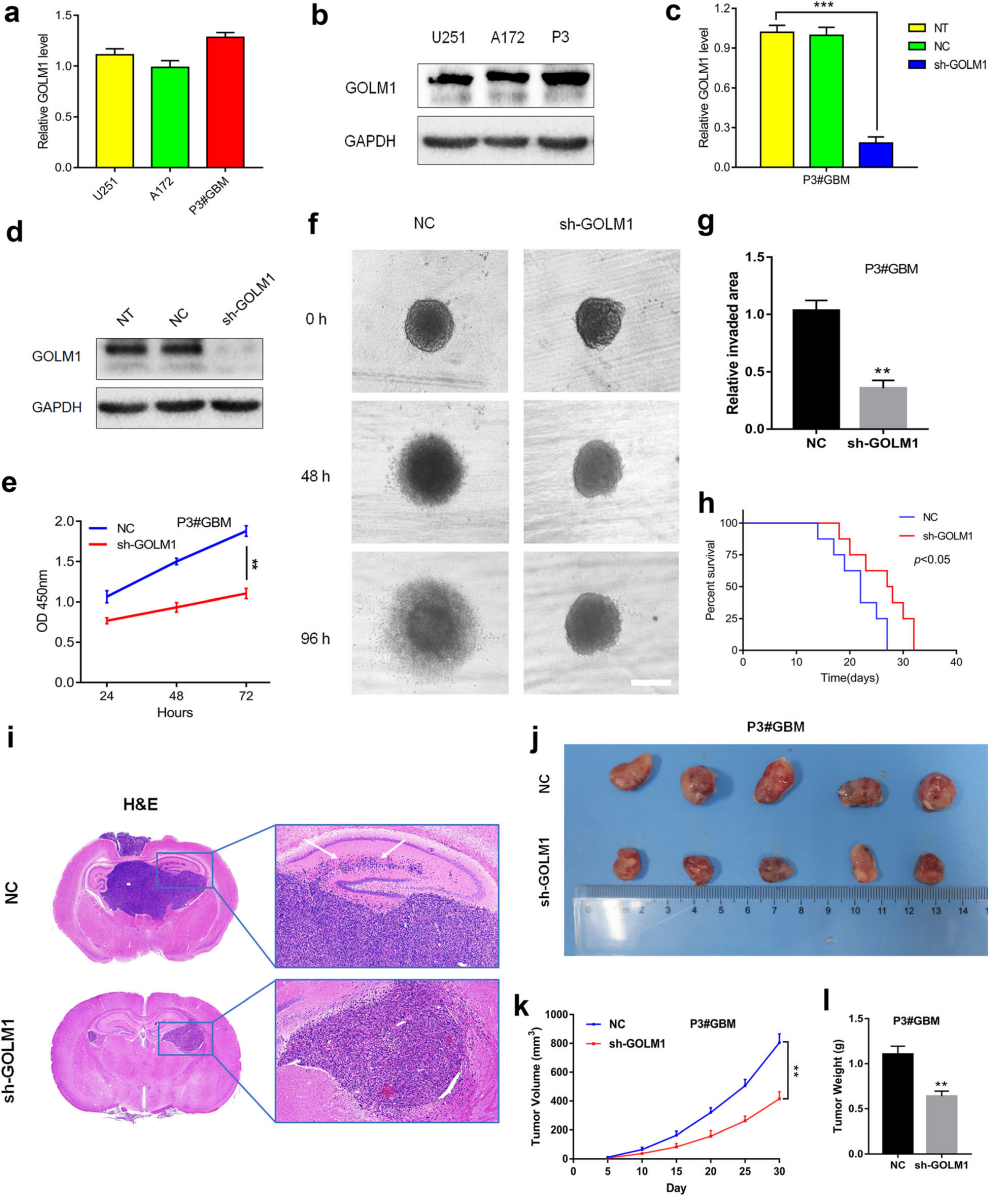
*GOLM1* knockdown inhibits glioma progression in P3#GBM cells in vitro and in vivo*.* Expression of GOLM1 in U251, A172 and P3#GBM cells was analyzed by **a** qRT-PCR and **b** western blot. Overexpression of GOLM1 in P3#GBM cells was confirmed by **c** qRT-PCR and **d** western blot analysis. **e** CCK8 assay for cell viability. **f** Representative images of invaded spheroids in 3D invasion assay for P3#GBM-NC and -sh-GOLM1 cells. Scale bar = 200 μm. **g** The area covered by invading cells was quantitated after 96 h of incubation. **h** Kaplan-Meier survival analysis of mice implanted with P3#GBM -NC (*n* = 8) and -sh-GOLM1 (*n* = 8) cells. The log-rank test was used to calculate *P*-values, which were <0.05. **i** Representative H&E images of intracranial tumors derived from P3#GBM -NC and -sh-GOLM1 cells. White arrows in zoomed image highlight tumor cells that have invaded to adjacent brain tissues. **j** Representative images of subcutaneous P3#GBM -NC and -sh-GOLM1 xenografts after surgical removal are also shown. **k** Tumor growth curves in nude mice from the P3#GBM -NC and -sh-GOLM1 groups. **l** Tumor weight from the P3#GBM -NC and -sh-GOLM1 groups. (**P < 0.01, ***P < 0.001)

Taking the heterogeneity of GBM into consideration, we investigated GOLM1 in P3#GBM which is an in vivo propagated primary GBM tumor cell line [19]. After comparison of GOLM1 level with U251 and A172 cells (Fig. 4a, b), P3#GBM cells were transfected with sh-GOLM1. The knockdown efficiency of GOLM1 was confirmed by qRT-PCR and western blot (Fig. 4c, d). Silencing of GOLM1 inhibited proliferation of P3#GBM cells which was indicated by the CCK-8 assay (Fig. 4e). Knockdown of GOLM1 also reduced the invaded area of P3#GBM spheroids in the 3D invasion model (Fig. 4f, g). We then sought to examine whether GOLM1 serves as an oncogene in P3#GBM-initiaed animal model. A longer survival time of tumor-bearing mice was observed in P3#GBM-sh-GOLM1 group (Fig. 4h). Furthermore, knockdown of GOLM1 dramatically inhibited invasion and growth of the tumor mass (Fig. 4i-l).

These results were consistent with a tumor promoting role for GOLM1 in U251 and A172 cell lines.

### GOLM1 overexpression promotes U87MG cells’ invasion and proliferation in vitro and in vivo

**Fig. 5:**
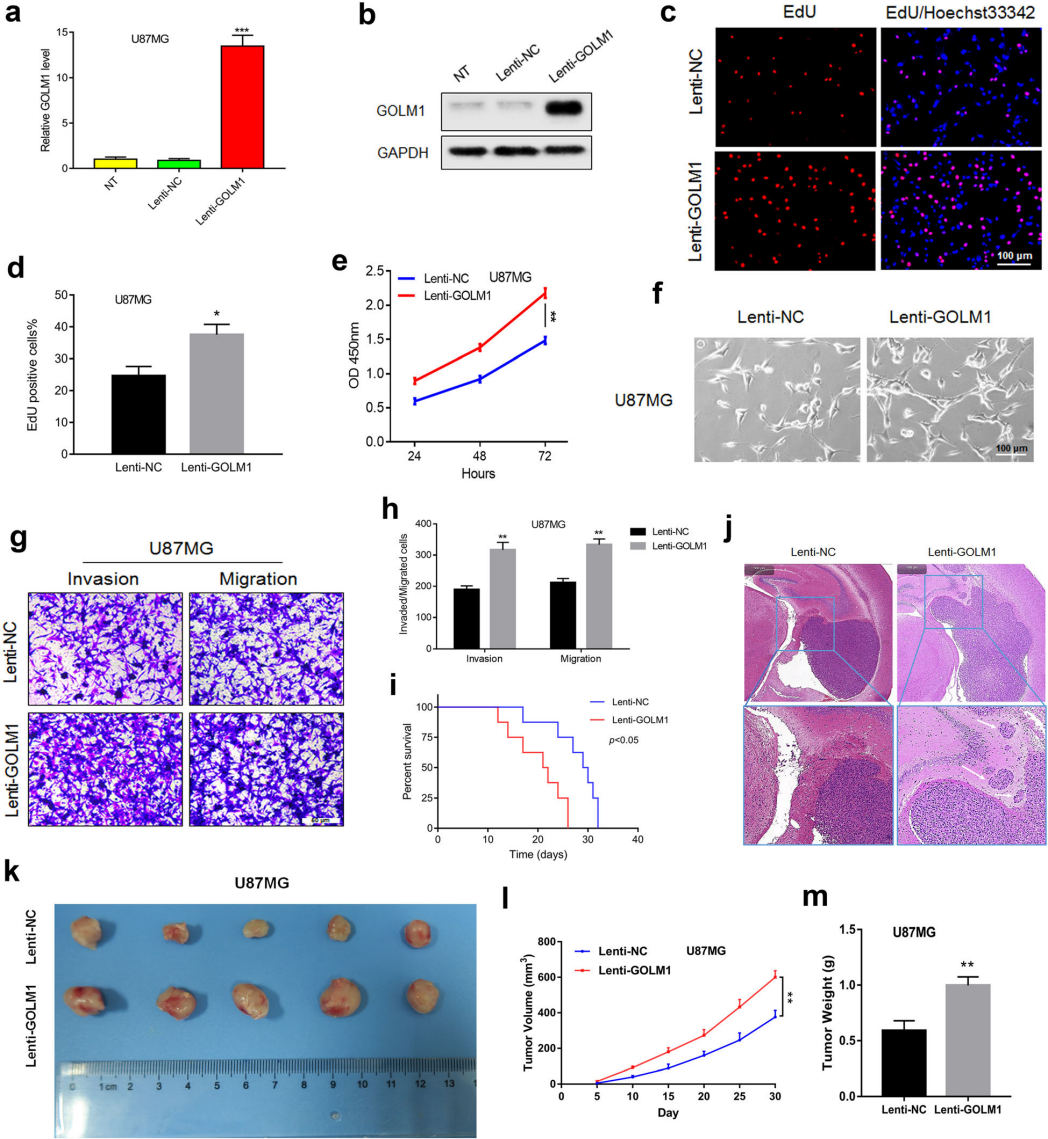
GOLM1 overexpression promotes U87MG cells’ invasion and proliferation in vitro and in vivo*.* Overexpression of GOLM1 in U87MG cells was confirmed by **a** qRT-PCR and **b** western blot analysis. **c** EdU assays for U87MG-Lenti-NC and -Lenti-GOLM1 cells. Scale bar = 100 μm. **d** Graphic representation of ratios of EdU positive U87MG- Lenti-NC and -Lenti-GOLM1 cells. Data are presented as the mean ± SEM. **e** Cell viability of U87MG-Lenti-NC or -Lenti-GOLM1 cells evaluated in the CCK8 assay. **f** Representative images of the morphology of U87MG- Lenti-NC and -Lenti-GOLM1 cells under bright field microscopy. Scale bar = 100 μm. **g** Representative images of Transwell assays performed with U87MG-Lenti-NC and -Lenti-GOLM1 cells after incubation for 24 h. Cells were fixed and stained with crystal violet. Scale bar = 50 μm. **h** Quantification of invaded and migrated cells in Transwell assays. Data are presented as the mean ± SEM. Scale bar = 50 μm. **i** Kaplan-Meier survival analysis of mice implanted with U87MG-Lenti-NC (*n* = 8) and -Lenti-GOLM1 (*n* = 8) cells. The log-rank test was used to calculate *P*-values, which were <0.05. **j** Representative H&E images of intracranial tumors derived from U87MG-Lenti-NC and -Lenti-GOLM1 cells. White arrows in the zoomed image highlight tumor cells that have invaded adjacent brain tissues. **k** Representative images of subcutaneous U87MG-Lenti-NC and -Lenti-GOLM1 xenografts after surgical removal are also shown. **l** Tumor growth curves in nude mice from the U87MG-Lenti-NC and -Lenti-GOLM1 groups. **m** Tumor weight from the U87MG-Lenti-NC and -Lenti-GOLM1 groups. Data are presented as the mean ± SEM. (*P < 0.05, **P < 0.01)

To examine the role of GOLM1 overexpression in human glioma development, we used U87MG cells which exhibited GOLM1 protein levels similar to NHA. We used a lentiviral construct for stable expression (Lenti-GOLM1). Increased GOLM1 was evident based on qRT-PCR and western blot analysis (Fig. 5a and b). The percentage of cells positive for EdU was increased (~ 20% to 40%) as well as cell viability (Fig. 5c–e). Thus, overexpression of GOLM1 led to increased proliferation and cell viability in U87MG cells. The results of clone-forming assays further confirmed these phenomenon (Additional file 2: Figure S2c).

U87MG-Lenti-GOLM1 cells also exhibited morphological differences that we thought could be associated with migration and invasion potential (Fig. 5f). Indeed, increased expression of GOLM1 was correlated with enhanced migration and invasion in Transwell assays relative to control cells (Fig. 5g and h).

Finally, to examine the impact of increased GOLM1 expression on glioma cell invasion and growth in vivo, modified U87MG cells were orthotopically implanted in nude mice. Survival time of U87MG-Lenti-GOLM1 relative to control tumor-bearing mice was decreased (Fig. 5i). On histologic examination, U87MG-Lenti-GOLM1 tumors exhibited increased invasion/migration as tumors no longer remained highly circumscribed (Fig. 5j). The promotion of GOLM1 on glioma growth was confirmed in the subcutaneous model, which was indicated by the increased tumor volume and tumor weight after upregulation of GOLM1(Fig. 5k-m).

### GOLM1 promotes human glioma progression through activation of AKT

**Fig. 6:**
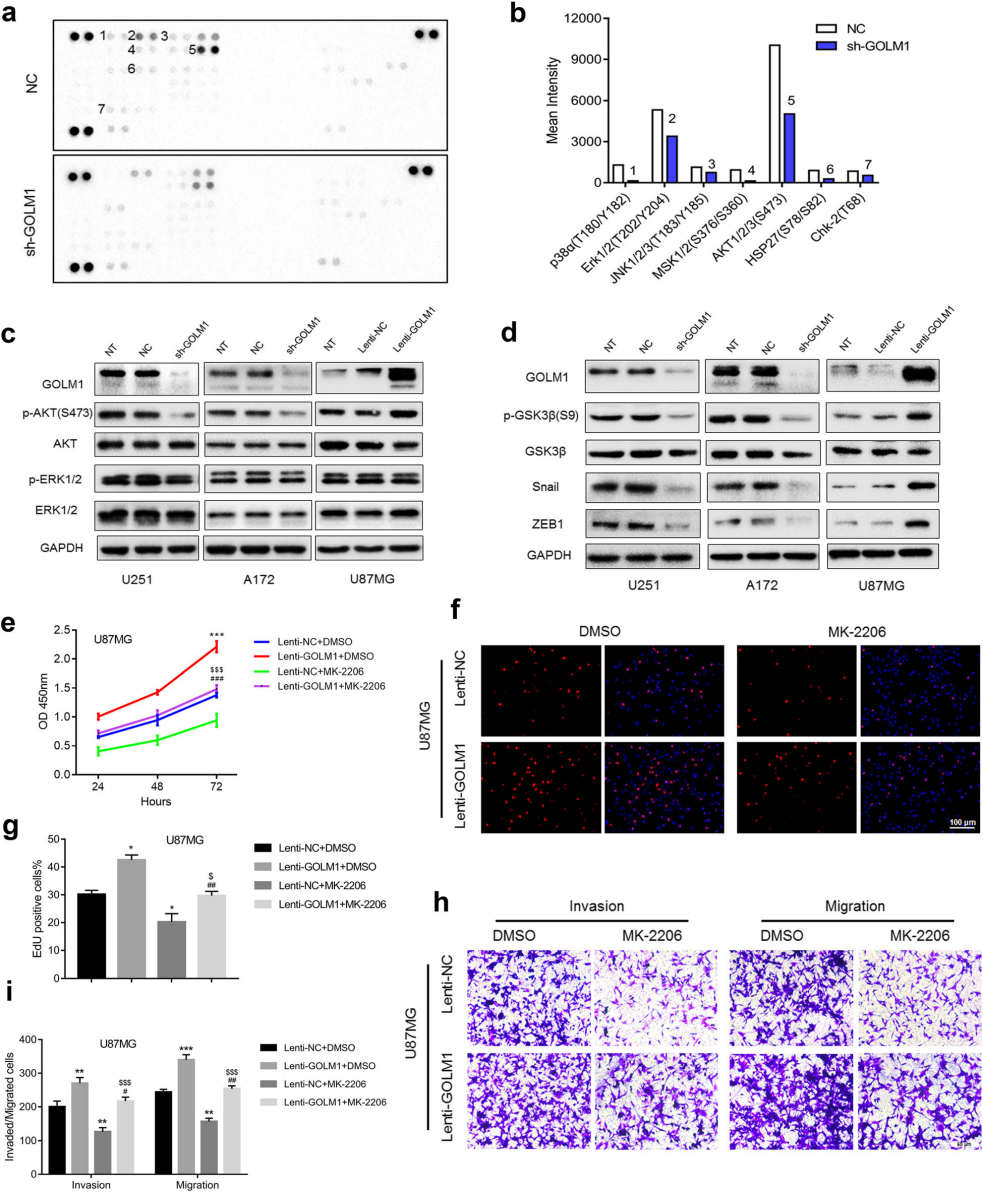
GOLM1 promotes human glioma progression through activation of AKT. **a** Image of phospho-kinase array performed with lysates prepared from U251-NC and -sh-GOLM1 cells. Spots with significant decreases in phosphorylation are numbered and quantification is shown in (**b**). **c** Western blot analysis of p-AKT (S473), AKT, p-ERK1/2, ERK1/2 in indicated cells. **d** Kinases and genes downstream of AKT in U251, A172 and U87MG cells were analyzed by western blot. U87MG-Lenti-NC and -Lenti-GOLM1 cells were treated with the AKT inhibitor MK-2206 (2 μM) or DMSO (vehicle control) and evaluated for **e** cell viability in the CCK8 assay and **f** cell proliferation in EdU (red) assays. Scale bar = 100 μm. **g** Graphic representation of ratios of EdU positive cells. Data are presented as the mean ± SEM. **h** Transwell migration and invasion assays were performed on U87MG-Lenti-NC and -Lenti-GOLM1cells with indicated treatment. **i** Quantification of invaded and migrated cells in Transwell assays after incubation for 24 h. Scale bar = 50 μm. (*P < 0.05 vs Lenti-NC + DMSO; **P < 0.01 vs Lenti-NC + DMSO; ***P < 0.001 vs Lenti-NC + DMSO; #*P* < 0.05 vs Lenti-GOLM1 + DMSO; ##*P* < 0.01 vs Lenti-GOLM1 + DMSO; ###*P* < 0.001 vs Lenti-GOLM1 + DMSO; $*P* < 0.05 vs Lenti-NC + MK-2206; $$$*P* < 0.001 vs Lenti-NC + MK-2206)

To illuminate the mechanisms underlying GOLM1 promotion in the development of human glioma, we used an antibody array to examine the phosphorylation status of 43 human phospho-kinases in lysates prepared from U251-NC and -sh-GOLM1 cells [20, 21]. In response to GOLM1 knockdown, we identified changes in the phosphorylation status of several phospho-kinases. Phosphorylation of p38 (T180/Y182), ERK1/2 (T202/Y204), JNK1/2/3 (T183/Y185), MSK1/2 (S376/S360), AKT1/2/3 (S473), HSP27 (S78/S82), Chk-2 (T68) decreased in U251-sh-GOLM1 cells (Fig. 6a and b). Based on previous studies [22–24] and the results of the antibody array (Fig. 6b), AKT and ERK signaling appear to be two of the main pathways driving glioma progression. Therefore, we examined whether phosphorylation of AKT and ERK might mediate GOLM1-induced proliferation, invasion, and migration in glioma. We first determined how phosphorylation of AKT and ERK in U251, A172, and U87MG might be regulated by GOLM1 levels. Phosphorylated AKT (Ser473) increased and decreased in parallel with changes in GOLM1 protein levels in modified cells. However, only a slight change was observed in phosphorylation of ERK with all GOLM1 constructs in all three cell lines (Fig. 6c, Additional file 3: Figure S3a).

We also examined the phosphorylation status of genes downstream of AKT, such as GSK3β, Snail and ZEB1 [25, 26], in response to altered levels of GOLM1. Phosphorylation of GSK3β and expression of ZEB1 and Snail decreased significantly with knockdown in U251-sh-GOLM1 and A172-sh-GOLM1 cells (Fig. 6d, Additional file 3: Figure S3b). These results were further examined in P3#GBM cells (Additional file 4: Figures S4a, 4b). In contrast, phosphorylation of these molecules increased with overexpression in U87MG-Lenti-GOLM1 cells (Fig. 6d, Additional file 3: Figure S3b).

To further examine the role of AKT in GOLM1 signaling, we exposed cells to an inhibitor of AKT (MK-2206) and evaluated proliferation and cell viability in U87MG-Lenti-NC and U87MG-Lenti-GOLM1 cells [27–29]. U87MG-Lenti-NC and -GOLM1 cells were treated with MK-2206 for 48 h. Proliferation and viability of cells was evaluated in CCK8 and EdU assays (Fig. 6e and f). MK-2206 attenuated glioma cell growth in U87MG cells. The enhanced cell viability of U87MG-Lenti-GOLM1 cells was also decreased under treatment with MK-2206 (Fig. 6e and g). We also found that the GOLM1-enhanced invasion and migration was suppressed by MK-2206 in U87MG-Lenti-GOLM1 cells (Fig. 6h and i). These results demonstrated that GOLM1 promoted proliferation, invasion, and migration through activation of AKT signaling in glioma cell lines.

### Expression of GOLM1 correlates with p-PDGFRα

**Fig. 7:**
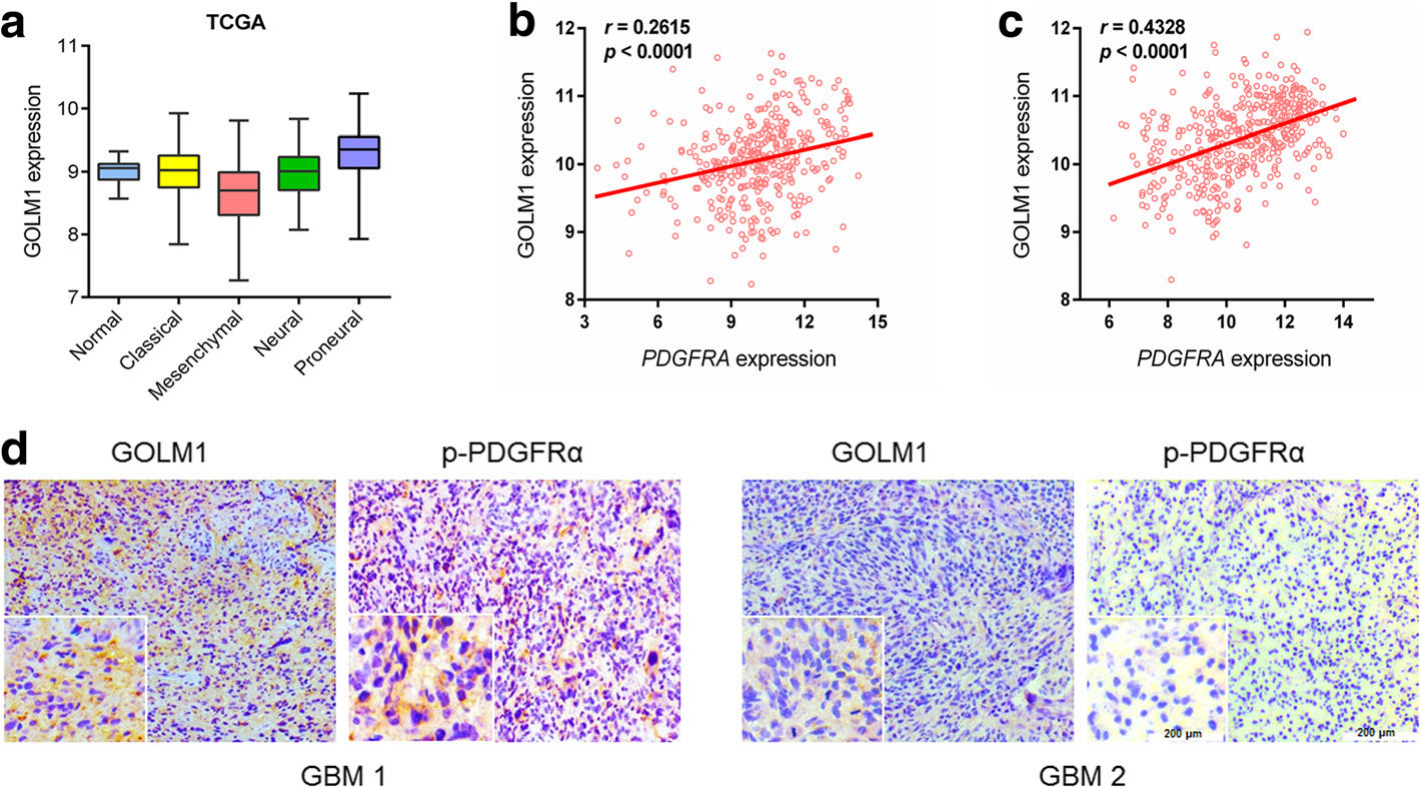
Expression of GOLM1 correlates with p-PDGFRα. **a** Relative expression levels of *GOLM1* in different glioma molecular subtypes (classical, mesenchymal, neural, and proneural) and normal brain tissue samples using the publicly available data from TCGA. Correlation between *GOLM1* and *PDGFRA* in gliomas determined using TCGA (**b**) and Rembrandt datasets **c**. The statistical significance of correlation was evaluated using a linear regression model (TCGA all glioma_cor_ = 0.261, *P* < 0.001; Rembrandt all glioma_cor_ = 0.433, *P* < 0.001). **d** IHC staining of p-PDGFRα and GOLM1 protein in primary human GBM samples. Representative images and magnified inset are labeled as GBM 1 and GBM 2. Scale bar = 200 μm

Molecular classification of GBM has led to the identification of four molecular subtypes, proneural, neural, classical, mesenchymal. Based on analysis of the publicly available TCGA data, higher levels of *GOLM1* tended to be associated with gliomas categorized as proneural (Fig. 7a). One of the major features of the proneural subtype is alterations in the gene for *PDGFRα* [30]. Thus, we examined the relationship between *PDGFRα* and *GOLM1* and found a positive correlation between the two genes in both TCGA and Rembrandt databases (Fig. 7b and c). To validate this association, we performed IHC for GOLM1 and p-PDGFRα on an independent cohort of GBM specimens obtained from our clinic (*n* = 29). In this cohort, increased GOLM1 was associated with increased p-PDGFRα (*P* = 0.014; Fig. 7d, Additional file 5: Table S1).

### GOLM1 may mediate PDGFA/PDGFRα signaling in A172 cells in vitro

**Fig. 8:**
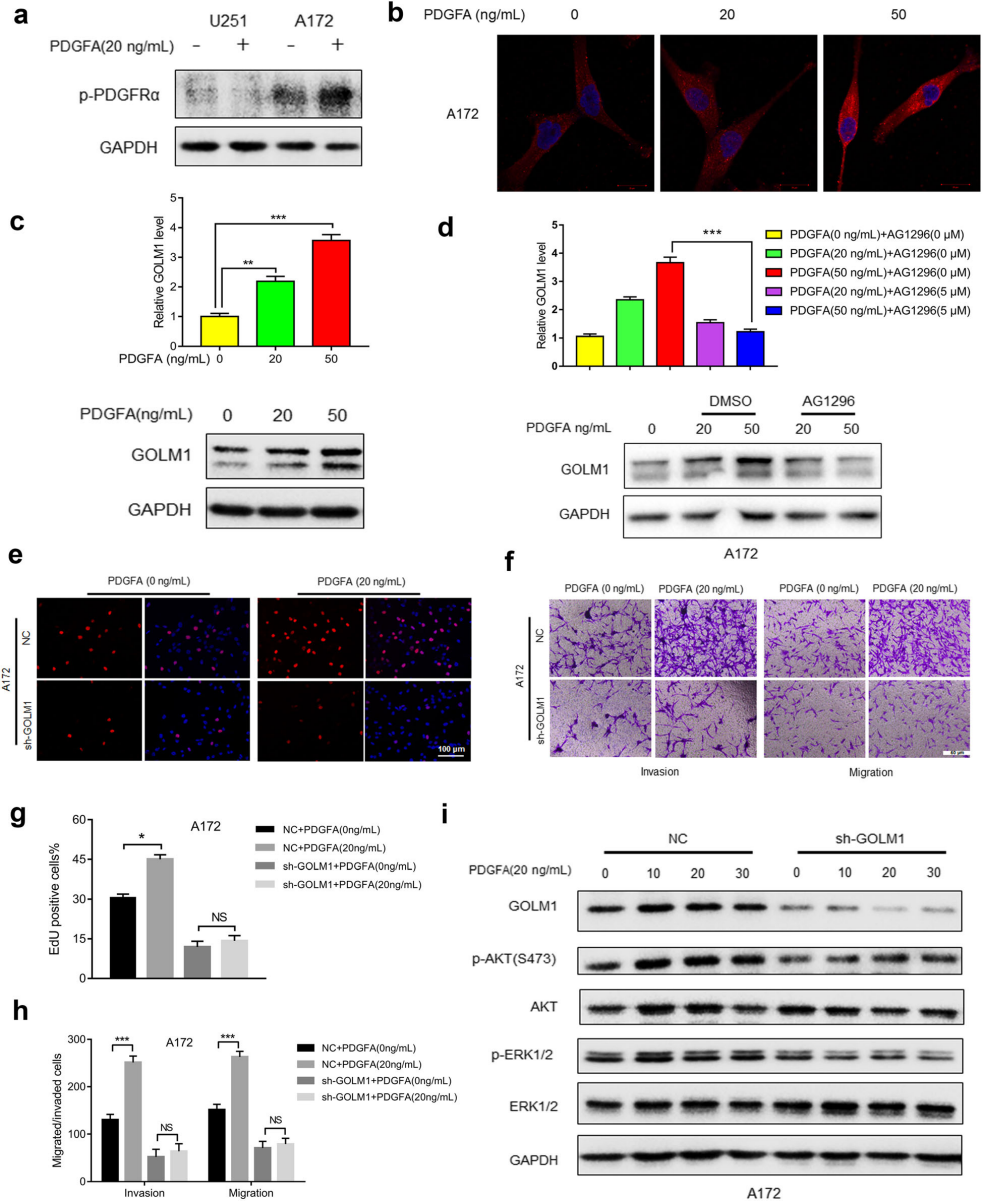
GOLM1 may mediate PDGFA/PDGFRα signaling in A172 cells in vitro*.* **a** Western blot analysis of p-PDGFRα in lysates prepared from PDGFA treated (20 ng/mL) and untreated U251 and A172 cells. PBS was used as the vehicle control. **b** IF staining of GOLM1 (red) in A172 cells treated with 0 ng/mL, 20 ng/mL and 50 ng/mL PDGFA for 24 h. Nuclei were labeled with DAPI (blue). Scale bar = 20 μm. **c** qRT-PCR (upper) and Western blot analysis (lower) of GOLM1 in lysates prepared from A172 cells treated with 0, 20, and 50 ng/mL PDGFA for 48 h. **d** qRT-PCR (upper) and Western blot analysis (lower) of GOLM1 in untreated cells or cells treated with increasing amounts of PDGFA in the presence of DMSO (vehicle control) or an inhibitor of PDGFRα AG1296 (5 μM) for 48 h. A172-NC and -sh-GOLM1 cells were treated with PBS (0 ng/mL PDGFA) as negative control or PDGFA (20 ng/mL) for 48 h. **e** EdU assays to evaluate cell proliferation under indicated treatment. Scale bar = 100 μm. **f** Representative images of Transwell migration and invasion assays performed in cells with indicated treatment. **g** Graphic representation of ratios of EdU positive cells under different treatments. Data are presented as the mean ± SEM. **h** Quantification of invaded and migrated cells in Transwell assays after incubation for 24 h. Data is presented as the mean ± SEM. Scale bar = 50 μm. **i** Western blot analysis of p-AKT (S473), AKT, p-ERK1/2 and ERK in A172-NC and -sh-GOLM1 cells after treatment with PDGFA (20 μg/mL) for 0, 10, 20, and 30 min. (NS, not significant; *P < 0.05, **P < 0.01, ***P < 0.001)

To investigate the functional relationship between PDGFRα and GOLM1, we examined GOLM1 protein levels in parental and modified glioma cells treated with PDGFA. We first exposed U251 and A172 cells with PDGFA and examined the phosphorylation status of PDGFRα by western blot [31]. Increased phosphorylation of PDGFRα occurred after treatment of A172 cells with recombinant PDGFA (20 ng/mL) for 48 h, but no change in p-PDGFRα was observed in U251 cells (Fig. 8a, Additional file 6: Figure S5a). We therefore used only A172 for further experiments with PDGFA. IF staining and western blot analysis was performed on A172 cells treated with increasing doses of PDGFA (20 ng/mL and 50 ng/mL) for 48 h to examine GOLM1 protein levels. Increased mRNA and protein levels of GOLM1 were observed in cells treated with a higher concentration of PDGFA (Fig. 8b and c, Additional file 6: Figure S5b). To further probe the relationship between GOLM1 and PDGFRα, we used a pharmacological inhibitor of PDGFRα, AG1296, to block receptor activity and examined GOLM1 levels on qRT-PCR and western blot analysis [32]. Increases in GOLM1 mRNA and protein in response to PDGFA (50 ng/mL) were inhibited by AG1296 treatment (Fig. 8d, Additional file 6: Figure S5c), indicating that activation of PDGFRα was critical for the modulation of GOLM1 by PDGFA.

Previous studies have demonstrated PDGFA/PDGFRα signaling contributes to the malignant behavior mainly through the activation of downstream genes AKT and ERK [31]. To assess whether GOLM1 also plays a role in PDGFA/PDGFRα-modulated activities in glioma, proliferation, invasion, and migration were first examined in parental A172 cells treated with PDGFA for 48 h. The percentage of EdU positive cells increased in response to PDGFA indicating enhanced proliferation (~ 30% to 45%; Fig. 8e, g). In addition, invasion and migration of A172 cells in Transwell assays increased dramatically (~ 3× and 2×, respectively; Fig. 8f, h). However, knockdown of GOLM1 almost completely abolished increases in these activities (Fig. 8e–h).

These results revealed a potential key role for GOLM1 in PDGFA/PDGFRα signaling. Therefore, we investigated whether GOLM1 mediated response between PDGFA/PDGFRα signaling and downstream genes AKT and ERK1/2. We performed western blot analysis with lysates prepared from A172-NC and A172-sh-GOLM1 cells after treatment with PDGFA for different time points and examined the phosphorylation status of AKT and ERK1/2. In control A172-NC cells, GOLM1, p-AKT and p-ERK1/2 were upregulated after treatment with PDGFA for 10 min (Fig. 8i, Additional file 6: Figure S5d). However, the activation of AKT, as well as ERK1/2, was not observed in A172-sh-GOLM1 cells (Fig. 8i, Additional file 6: Figure S5d). These results indicated that GOLM1 may play a role in mediating PDGFA/PDGFRα signaling.

## Discussion

**Fig. 9:**
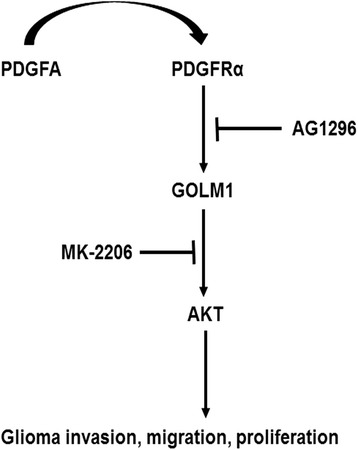
A working model of PDGFA/PDGFRα signaling mediated by GOLM1 in glioma migration, invasion and proliferation

Molecular therapy has emerged as a promising strategy to improve treatment efficiency and prolong survival time of cancer patients. Identification of novel therapeutic targets is critical for the design of more effective tumor specific strategies and requires a combination of molecular and functional studies [33–35]. In this work, we identified GOLM1 as a potential target of glioma molecular therapy. We found GOLM1 mRNA and protein levels were increased in GBM compared to normal brain tissues. Based on these findings, we investigated the function of GOLM1 in glioma and found that it was significantly associated with glioma invasion and migration, as well as proliferation. Furthermore, we found that AKT activation was the key element in GOLM1-induced glioma progression. Finally, we demonstrated that GOLM1 acts as downstream gene of PDGFA/PDGFRα signaling (Fig. 9).

These results in glioma are supported by similar findings in many other human cancers. GOLM1 expression is increased in aggressive tumor types [7–10]. Based on previous studies [7–10], we examined the role of GOLM1 in the proliferation, invasion, and migration of glioma cell lines and observed that the protein promotes these properties in glioma, not only in vitro but also in vivo. Notably, aggressive growth and infiltrative nature are two main features of glioma progression [34–36]. Phosphorylation of AKT at S473 was identified as an underlying molecular event mediating GOLM1 activity, and we confirmed the involvement of AKT activation by analyzing protein/phosphorylated levels of downstream targets of the kinase, including ZEB1, GSK3β and Snail. In addition, we examined GOLM1 function in the presence of an inhibitor of AKT phosphorylation, MK-2206. AKT mediates many key cellular processes including proliferation, cell survival, metabolism, growth and angiogenesis in many types of human cancer, and many of these processes are performed through the activation of a range of downstream genes several of which are involved in molecular networks in glioma progression. Previous studies have demonstrated, for example, that Snail and ZEB1 transcription factors are involved in the process of epithelial–mesenchymal transition (EMT) which is a cellular alteration that confers a more invasive phenotype on cells [31, 37, 38]. Phosphorylation of GSK-3β by AKT is also thought to be a mechanism underlying the promotion of cell proliferation [39, 40]. Although we found that GOLM1 accelerates glioma progression through activation of AKT and its downstream effectors, our study has not fully illuminated the precise function of GOLM1 in AKT-related molecular networks.

Finally, we found that GOLM1 is regulated by PDGFA/PDGFRα signaling and has a relatively central role mediating response through this pathway. As a classical growth factor, PDGFA can activate SHP-2/ERK and PI3K/AKT pathways through binding to its specific receptor, PDGFRα [31]. This process can lead to a more aggressive phenotype in many human cancers including glioma [41]. Focal amplifications of the locus at 4q12 harboring PDGFRA have been observed in almost all molecular subtypes of human glioma but more frequently in the proneural subtype [30, 42]. In addition, PDGFRα and its main ligand PDGFA are key mediators of glial cell proliferation, mainly oligodendrocytes, which have a critical role in normal development of central nervous system [43]. Many clinical trials evaluating the efficacy of anti-PDGFRA therapies on human gliomas are in development [44–46]. We have demonstrated that GOLM1 has a linear correlation with p-PDGFRα and therefore maintains a critical position between PDGFA/ PDGFRα and its downstream genes. Therefore, our results may help to provide the basis for a more specific therapeutic regimen for the patients of proneural subtype glioma.

## Conclusions

In summary, GOLM1 facilitates proliferation, invasion, and migration of human glioma cell lines potentially through the activation of AKT. Furthermore, we demonstrated that PDGFA/ PDGFRα upregulates GOLM1, and that GOLM1 acts as a key component in PDGFA/ PDGFRα-mediated glioma progression. These results raise the possibility that targeting of GOLM1 may represent a promising strategy for the treatment of human gliomas.

## Additional files

Additional file 1: Figure S1. Expression of GOLM1 were analyzed in normal brain tissues (*n* = 4), WHO II gliomas (*n* = 4) and WHO III-IV gliomas (*n* = 11). (TIFF 682 kb)

Additional file 2: Figure S2. (a-b) Representative images and graphic representation of colony forming assays for U251- and A172-NC or sh-GOLM1 cells. (c) Representative images and graphic representation of colony forming assays for U87MG- Lenti-NC or -Lenti-GOLM1 cells. Data are presented as the mean ± SEM. (TIFF 5314 kb)

Additional file 3: Figure S3. ImageJ was introduced to assess the western blot results in in Fig. 6c (a) and 6d (b). Data are presented as the mean ± SEM. (TIFF 2306 kb)

Additional file 4: Figure S4. (a) Kinases and genes downstream of AKT in P3#GBM cells were analyzed by western blot. (b) ImageJ was introduced to assess the western blot results in (a). Data are presented as the mean ± SEM. (TIFF 2694 kb)

Additional file 5: Table S1. Association of p-PDGFRα with GOLM1 protein levels in primary human GBM samples (*n* = 29). (DOCX 14 kb)

Additional file 6: Figure S5. ImageJ was introduced to assess the western blot results in in Fig. 8a (a), 8c (b), 8d (c) and 8i (d). Data are presented as the mean ± SEM. (TIFF 2057 kb)

## Abbreviations

AKT (PKB): Protein Kinase B
ERK: Extracellular Regulated protein Kinases
GSK3β: Glycogen Synthase Kinase 3 Beta
ZEB1: Zinc Finger E-Box Binding Homeobox 1
