# Supplementary figures and images for: PDGFA/PDGFRα-regulated GOLM1 promotes human glioma progression through activation of AKT
Source: J Exp Clin Cancer Res. 2017 Dec 28;36:193. doi: 10.1186/s13046-017-0665-3 (PMC5745991; doi:10.1186/s13046-017-0665-3)

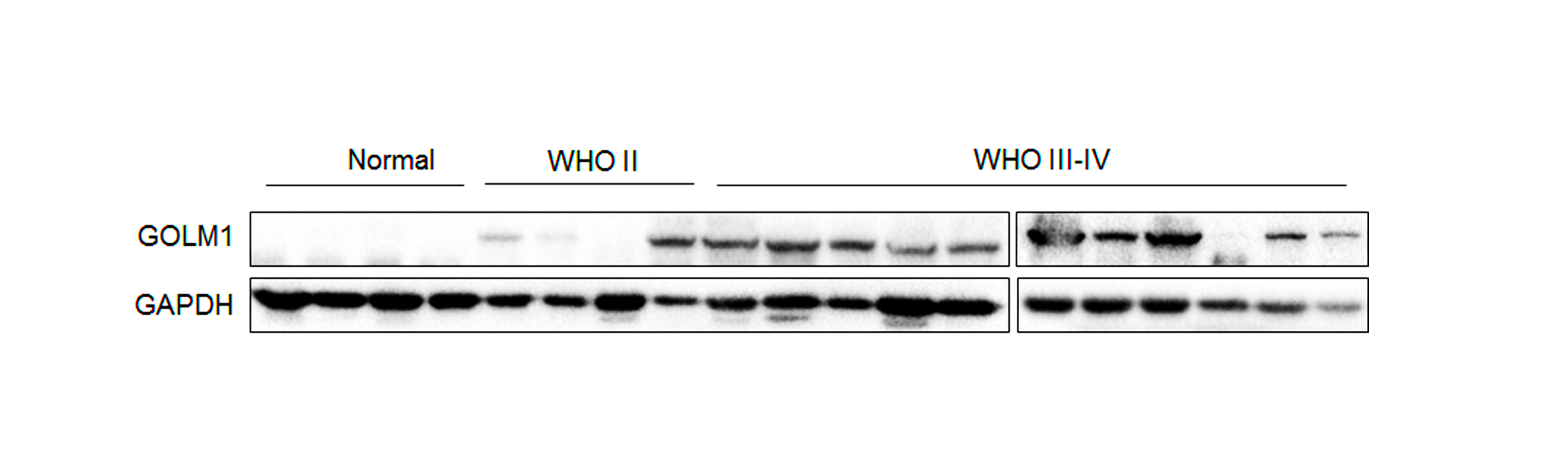

Supplement: Supplementary file 1 — Expression of GOLM1 were analyzed in normal brain tissues (n = 4), WHO II gliomas (n = 4) and WHO III-IV gliomas (n = 11). (TIFF 682 kb) [file 13046_2017_665_MOESM1_ESM.tif]

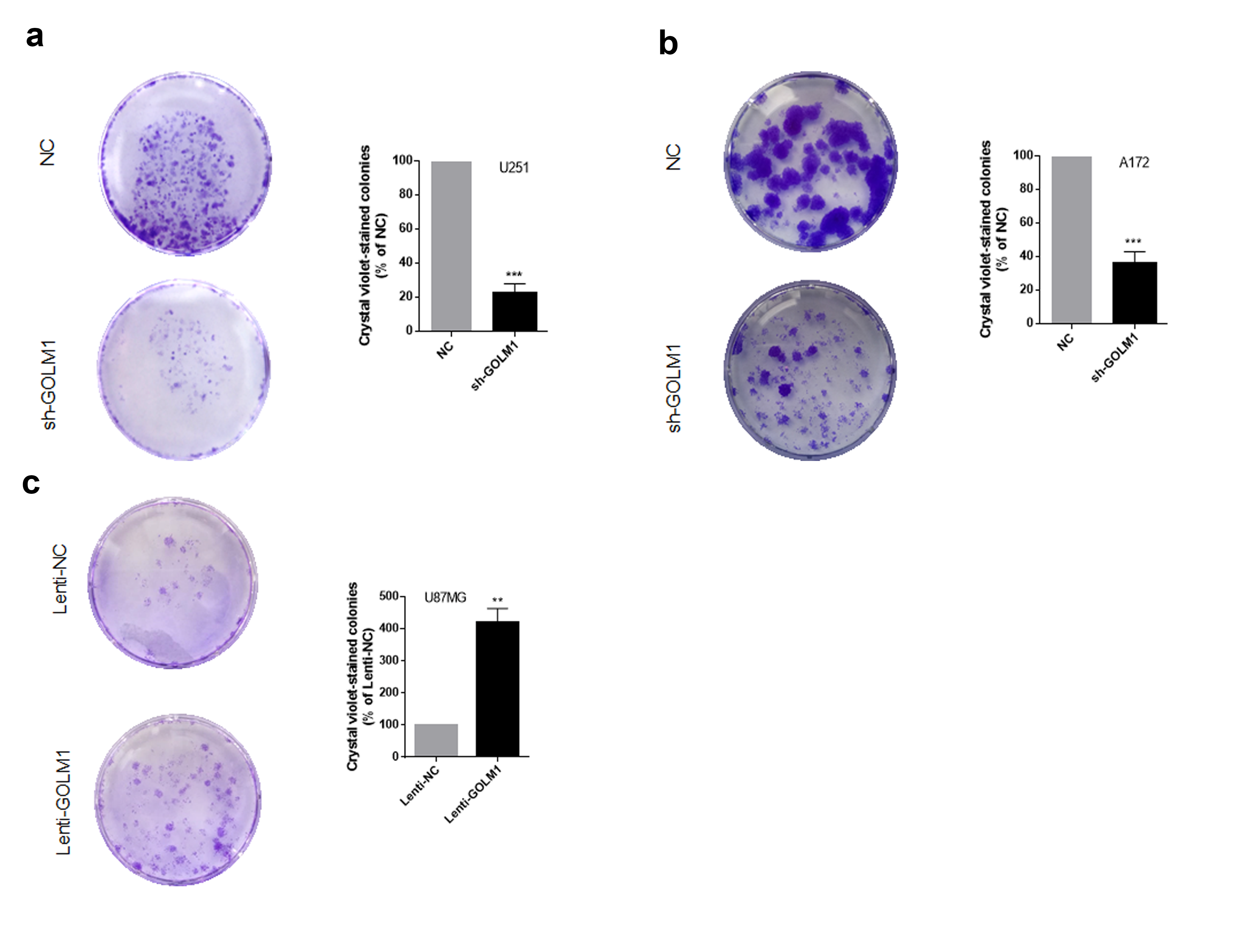

Supplement: Supplementary file 2 — (a-b) Representative images and graphic representation of colony forming assays for U251- and A172-NC or sh-GOLM1 cells. (c) Representative images and graphic representation of colony forming assays for U87MG- Lenti-NC or -Lenti-GOLM1 cells. Data are presented as the mean ± SEM. (TIFF 5314 kb) [file 13046_2017_665_MOESM2_ESM.tif]

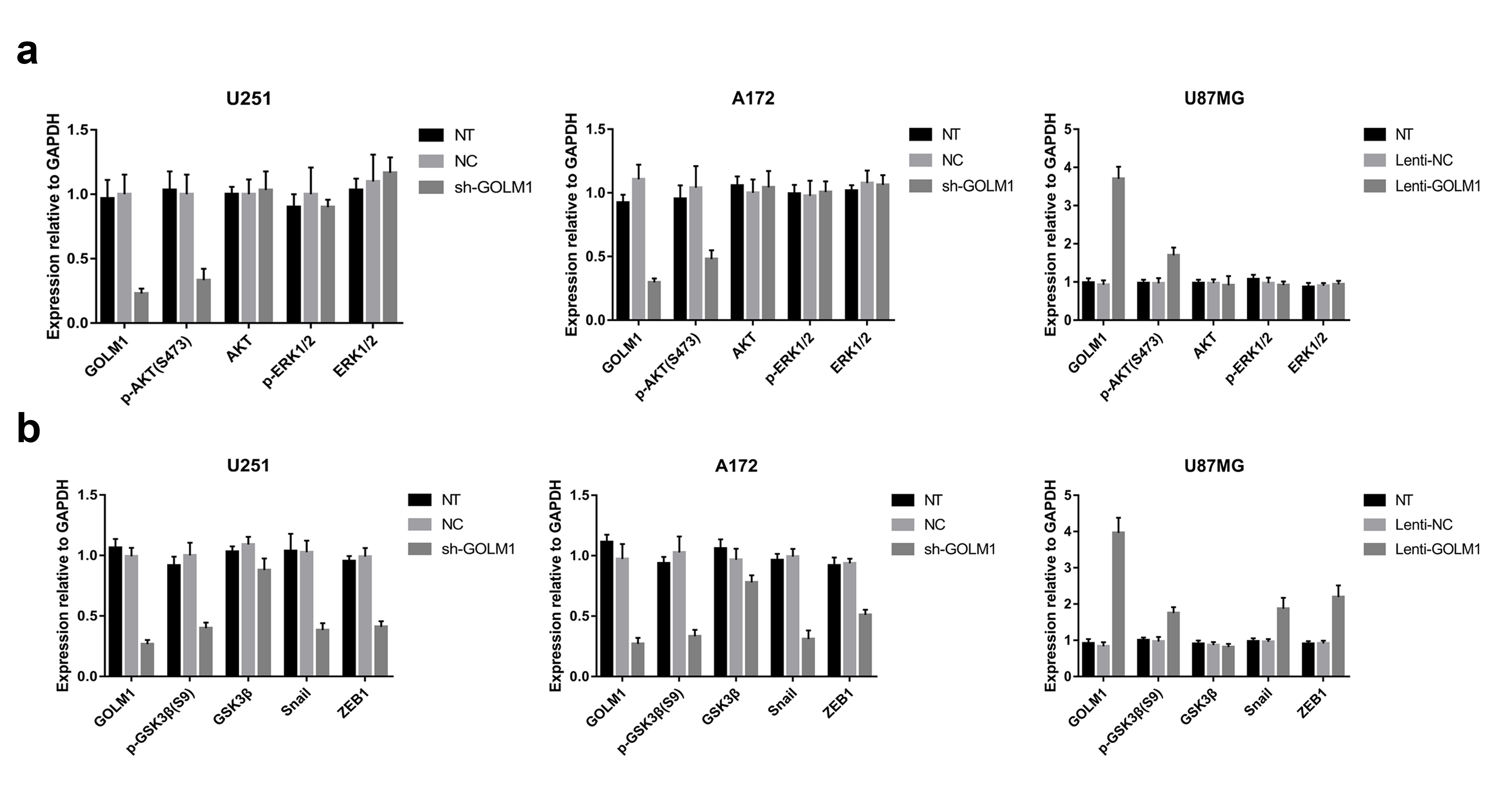

Supplement: Supplementary file 3 — ImageJ was introduced to assess the western blot results in in Fig. 6c (a) and 6d (b). Data are presented as the mean ± SEM. (TIFF 2306 kb) [file 13046_2017_665_MOESM3_ESM.tif]

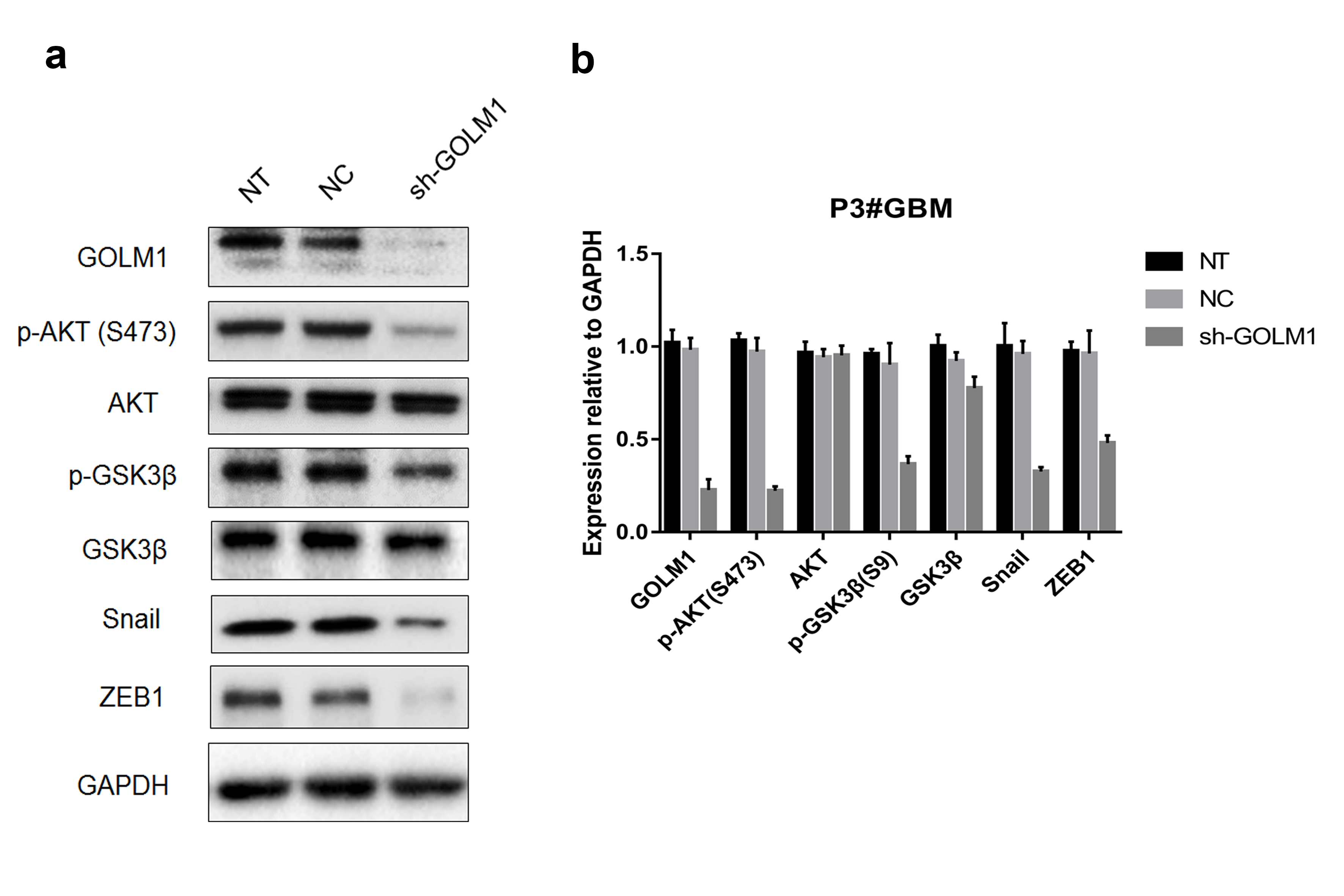

Supplement: Supplementary file 4 — (a) Kinases and genes downstream of AKT in P3#GBM cells were analyzed by western blot. (b) ImageJ was introduced to assess the western blot results in (a). Data are presented as the mean ± SEM. (TIFF 2694 kb) [file 13046_2017_665_MOESM4_ESM.tif]

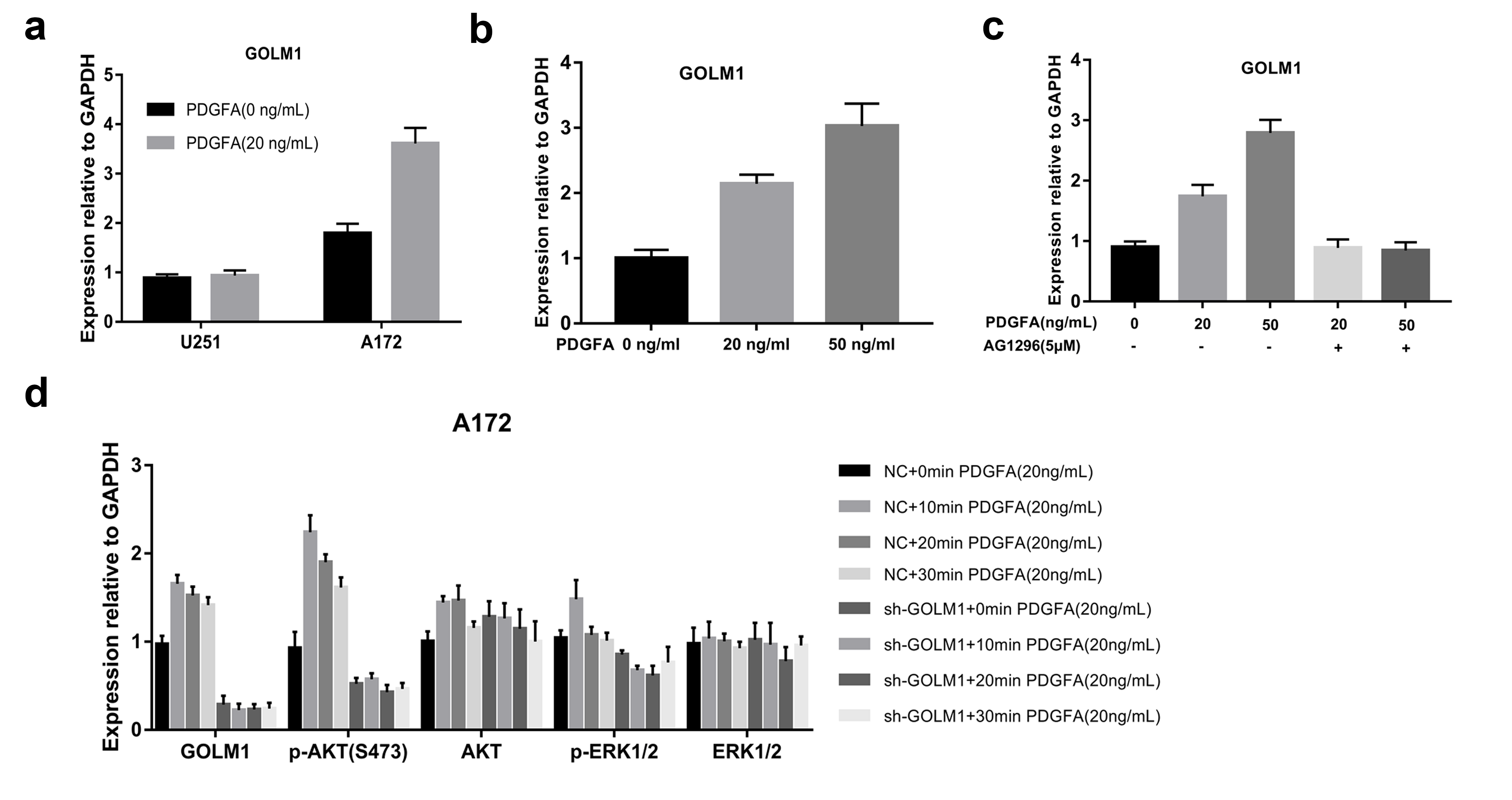

Supplement: Supplementary file 6 — ImageJ was introduced to assess the western blot results in in Fig. 8a (a), 8c (b), 8d (c) and 8i (d). Data are presented as the mean ± SEM. (TIFF 2057 kb) [file 13046_2017_665_MOESM6_ESM.tif]
