# Supplementary material for: PDGFA/PDGFRα-regulated GOLM1 promotes human glioma progression through activation of AKT
Source: J Exp Clin Cancer Res. 2017 Dec 28;36:193. doi: 10.1186/s13046-017-0665-3 (PMC5745991; doi:10.1186/s13046-017-0665-3)
Supplement: Supplementary file 5 — Association of p-PDGFRα with GOLM1 protein levels in primary human GBM samples (n = 29). (DOCX 14 kb) [file 13046_2017_665_MOESM5_ESM.docx]

|  | | GOLM1 expression | | *P* value |
| --- | --- | --- | --- | --- |
|  |  | Weak | Strong |  |
| p-PDGFRα expression | Weak | 10 | 3 | 0.014 |
|  | Strong | 5 | 11 |  |

**Table S1. Association of p-PDGFRα with GOLM1 protein levels in primary human GBM samples (*n* = 29)**
